# Determining Tube Theory Parameters by Slip-Spring Model Simulations of Entangled Star Polymers in Fixed Networks

**DOI:** 10.3390/polym11030496

**Published:** 2019-03-14

**Authors:** Jing Cao, Zuowei Wang, Alexei E. Likhtman

**Affiliations:** School of Mathematical, Physical and Computational Sciences, University of Reading, Reading RG6 6AX, UK

**Keywords:** entangled polymers, slip-spring model, slip-link, tube theory, arm retraction dynamics, first passage time, primitive path, relaxation correlation function

## Abstract

Dynamical properties of branched polymer melts are determined by the polymer molecular weights and architectures containing junction points. Relaxation of entangled symmetric star polymers proceeds via arm-retraction and constraint release (CR). In this work, we investigate arm-retraction dynamics in the framework of a single-chain slip-spring model without CR effect where entanglements are treated as binary contacts, conveniently modeled as virtual “slip-links”, each involving two neighboring strands. The model systems are analogous to isolated star polymers confined in a permanent network or a melt of very long linear polymers. We find that the distributions of the effective primitive path lengths are Gaussian, from which the entanglement molecular weight $N_{e}$, a key tube theory parameter, can be extracted. The procured $N_{e}$ value is in good agreement with that obtained from mapping the middle monomer mean-square displacements of entangled linear chains in slip-spring model to the tube model prediction. Furthermore, the mean first-passage (FP) times of destruction of original tube segments by the retracting arm end are collected in simulations and examined quantitatively using a theory recently developed in our group for describing FP problems of one-dimensional Rouse chains with improbable extensions. The asymptotic values of $N_{e}$ as obtained from the static (primitive path length) and dynamical (FP time) analysis are consistent with each other. Additionally, we manage to determine the tube survival function of star arms μ(t), or equivalently arm end-to-end vector relaxation function ϕ(t), through the mean FP time spectrum τ(s) of the tube segments after careful consideration of the inner-most entanglements, which shows reasonably good agreement with experimental data on dielectric relaxation.

## 1. Introduction

Dynamics of entangled polymer melts have been predominantly described by theories based on the tube model. de Gennes [1], Doi and Edwards [2] introduced the concept of confining tube to account for the topological constraints on the motion of a target chain arising from the uncrossability of surrounding chains in concentrated polymer solutions and melts. Original tube theories developed for describing stress relaxation of entangled linear chain systems following a step strain focus on three main relaxation mechanisms, namely contour length fluctuation (CLF), constraint release (CR) and reptation, by which a chain escapes from its original confining tube and forgets about the anisotropic conformation induced by the step strain to release the stored stress. Tube-based theories have provided reasonably good predictions on linear and nonlinear viscoelastic behaviors of monodisperse linear polymers with high molecular weights [3,4,5,6,7,8], with the entanglement molecular weight $N_{e}$ and the Rouse relaxation time $\tau_{e}$ of an entanglement strand as input parameters.

Relaxation of entangled branched polymers undergoes a hierarchical way, starting from the retraction of the outermost branch arms and proceeding to inner layers until the cores of the molecules. For the simplest case of symmetric star polymers in a fixed network, the pioneering work of Pearson and Helfand [9] treated their relaxation process as an one-dimensional (1D) arm retraction problem. The characteristic relaxation time or first-passage (FP) time of a specific tube segment is determined by the time when the arm free end reaches it for the first time. For simplification, the retracting arm was modeled as one single particle which carries the friction experienced by the entire arm and fluctuates under an entropic potential $U\left(s\right)=\frac{3N}{2N_{e}}s^{2}$, where *N* is the number of beads in the arm and *s* is the fractional retraction distance along the primitive path (PP). The mean FP time τ(s) grows exponentially with *s* as $\tau \left(s\right)=\tau_{0}\exp\left[U\left(s\right)\right]$ where $\tau_{0}$ is related to the inverse “attempt frequency”. For concentrated solutions and melts of star polymers, the predictions of this theory showed large discrepancy from experimental measurements due to the neglect of CR effects. Ball and McLeish [10] treated the CR effects in a self-consistent way by assuming the relaxed arm segments as a solvent for the unrelaxed materials, which is generally termed as “dynamic tube dilation” (DTD). Later, Milner and McLeish [11] improved the theory for predicting mean FP time of arm retraction by solving the Kramers’ problem of one bead linked to the origin through a harmonic spring. After combining with the contribution of early time fluctuations of the arm free end, the theory predicts the stress loss modulus $G^{\prime \prime }$ of symmetric star polymer melts in good agreement with experiments [12]. However, the Milner–McLeish theory encountered difficulties in quantitatively predicting the dielectric or arm end-to-end vector relaxation functions of symmetric stars [12] and in describing the rheological behaviors of asymmetric stars with different short arm lengths by using a single set of model parameters [13]. Such inadequacies inspire further understanding of relaxation mechanisms in star polymer melts as well as examining the DTD picture during the last decade.

Recently, we demonstrated that the Pearson–Helfand theory and subsequently the Milner–McLeish theory without constraint release oversimplified the arm retraction problem by only considering the slowest relaxation mode of the arms [14]. Instead, the entangled arms should be represented by 1D Rouse chains moving in the confining tubes because the fast relaxation modes play an important role in the tube segment destruction. The arm retraction dynamics can be considered as a multi-dimensional Kramers’ problem in the normal space [15]. The position of the arm free end in real space is transformed to a hyperplane in the normal space such that the original FP problem turns to a FP problem of a fluctuating particle reaching an absorbing hyperplane in multi-dimensional space under certain potential landscape. The dynamics is projected onto the most probable trajectory between the origin and the absorbing hyperplane, termed as “minimal action path” (MAP). The preceding mean FP time τ(s) can be derived based on the conventional Kramers’ solution along the MAP,

$$\tau \left(s\right)∼\frac{1}{s^{3}}\exp\left(\frac{3Zs^{2}}{2}\right),$$

where $Z=N/N_{e}$ is the number of entanglements per arm. Calculations using this so-called large deviation theory show that the Milner–McLeish theory without CR overestimates the FP time by a factor of 10 or more.

For examining the assumptions made in different theoretical models and validating their predictions, molecular dynamics (MD) simulations based on bead-spring models have been widely accepted as one of the most suitable candidates because they allow the direct visualization of the conformations and trajectories of individual polymers as well as their topological interactions with neighboring chains, which are essential for understanding the microscopic relaxation mechanisms underlying entanglement dynamics [16,17,18,19,20,21,22,23,24,25,26,27,28,29,30,31]. However, the high demand for computational power has limited MD simulations to systems with relatively low polymer molecular weights and smaller system sizes in comparison with real experimental setups. The intrinsically slow relaxation of entangled branched polymers, such as star, H-, comb and Cayley-tree polymers, even further compresses the parameter space that MD simulations are able to explore confidently. Therefore, more coarse-grained (CG) simulation models, such as the slip-spring and slip-link models [32,33,34,35,36,37,38,39,40,41], have been developed to bridge the gap between tube theories and MD simulations. One essential hypothesis made in these models is the binary contact picture between entangled polymers. This picture has been qualitatively validated by analyzing the persistent close-contacts between pairs of neighboring polymer strands in both linear and star polymer melts obtained from MD simulations based on the standard bead-spring model [31]. Under this picture, $N_{e}\left(\phi \right)$ in concentrated polymer solutions appears to scale linearly with 1/ϕ where ϕ is the polymer volume fraction [42], which is also supported by topological analysis of ring polymer melts in mesoscopic MD simulations [43] and entanglement analysis of Polyethylene networks and melts in atomistic MD simulations [44]. We will thus employ the single-chain slip-spring model originally developed by Likhtman [21,24,31,32,45] to examine the theoretical models for describing the entanglement dynamics of star polymers [9,10,11,14]. The main focus of the current work will be the dynamics of symmetric stars in a permanent network, aiming at evaluating the predictions of the Pearson–Helfand theory [9] and the large deviation theory [14] and accordingly extracting the important tube theory parameters, including $N_{e}$. This study is carried out by taking advantage of the slip-spring model which can switch on and off constraint release without affecting other relaxation mechanisms.

Zhu et al. recently presented an efficient simulation algorithm by combining the single-chain slip-spring model with the forward flux sampling method for studying arm retraction dynamics of strongly entangled symmetric stars [46]. In the absence of CR effects, the arm terminal relaxation times $\tau_{d}$ and the dielectric and stress relaxation functions are reported for star polymers with arm lengths up to 16 entanglements, which are generally inaccessible to direct simulation methods. In that work, it was pointed out that, in the systems without CR, the arm terminal relaxation times and the $N_{e}$ values obtained by fitting the simulation data to theoretical models may have some quantitative sensitivity to the discrete destruction of tube segments in the slip-spring model, especially in relation to the release of the innermost slip-link or tube segment. Considering that this is a common problem for all discrete models, we will propose a numerical solution to this issue.

In experiments, dynamics of star polymers in nearly non-CR environment have been studied by Matsumiya et al. using star-branched polyisoprene (PI) probes diluted in a matrix of much longer linear PI chains [47]. The relaxation of the star probes was shown to be retarded and broadened due to the quenching of constraint release effects, as made evident by the coincidence of the frequency dependence of their dielectric and viscoelastic losses. Quantitative analysis of the retardation magnitude of the probes within the context of the tube model indicated that the conventional DTD picture [10,11] is not sufficient to explain the relation between the viscoelastic relaxation and dielectrically evaluated survival fraction of the dilated tube of PI stars in monodisperse bulk. A different mechanism was suggested where arm retraction of star polymers in the bulk proceeds along the longitudinally partially dilated tube that wriggles in the laterally partially dilated tube. We show in this work that the single-chain slip-spring model without CR can produce simulation results on the dielectric losses of star polymers in reasonably good agreement with experimental data on the probe PI stars [47]. Its predictions on the arm retraction dynamics in the absence of CR can thus be compared with simulation results obtained from the same slip-spring model but for monodisperse melts [31] as well as those from multichain slip-spring/slip-link model [48,49] and more detailed molecular dynamics [31,50,51,52] simulations to provide microscopic insights into the proposed constraint release mechanisms.

The rest of this paper is organized as follows. First, the details of the single-chain slip-spring model are summarized in Section 2. In Section 3, we will construct the primitive paths of the confining tubes of star arms in two different ways. In Section 4, the tube step length *a* and the entanglement molecular weight $N_{e}$ will be determined by probing static properties of the primitive paths. The obtained $N_{e}$ values based on different constructions of the primitive paths are found to be in very good agreement with each other. Furthermore, they are also consistent with that estimated by mapping the slip-spring model simulation results on the middle monomer mean-square displacements of linear chains to tube theory prediction. In Section 5, we will introduce an efficient and convenient algorithm to measure the mean FP time τ(s) of tube segment destruction on the fly. Our results suggest that in the systems without CR the FP times τ(s) of tube segment destruction do have exponential distributions close to the branch points, just as predicted by earlier tube theories. A reasonable $N_{e}$ value can be determined by comparing the τ(s) results with predictions of the large deviation theory [14]. In the end, the tube survival function μ(t), or equivalently the stress relaxation function in the current study, will be recovered from τ(s) and shown to have very good agreement with direct measurement of the arm end-to-end relaxation function ϕ(t). A conclusion section follows.

## 2. Slip-Spring Model

In the single-chain slip-spring model we used, the star arms are represented by Rouse chains in three-dimensional space, each consisting of N+1 beads linked by *N* harmonic springs [21,24,31,32,45]. The average spring bond length $b_{3D}$, the friction coefficient of each bead $\xi_{3D}$, the temperature $k_{B}T$ and the slip-spring unit time scale are all set to unity. The subscript 3D used here is to distinguish from the one-dimensional Rouse model parameters introduced later. The confinement due to entanglements are modeled in a discrete manner by a set of virtual springs, each having one end connected to the Rouse chain by a slip-link, while the other end (anchor point) fixed in space [32,45]. This approach effectively treats entanglements as binary contacts between two arms such that each slip-link is paired with another slip-link sitting on an arm from a different star polymer in the system.

Apart from the standard parameters of the Rouse model [2], there are two other adjustable parameters related to entanglements, namely the average number of monomers in between two neighboring slip-links, $N_{e}^{ss}$, and the average number of monomers per virtual spring $N_{s}^{ss}$. It is important to note that $N_{e}^{ss}$ is not necessarily equal to the entanglement length $N_{e}$ as defined in the tube theory. The values of $N_{e}^{ss}$ and $N_{s}^{ss}$ can be adjusted for controlling the intensity of entanglements. The trajectories of the Rouse monomers are determined by the Langevin equations of motion [2]. In the original slip-spring model [32,53], the slip-links are assumed to travel continuously along the straight lines between adjacent monomers and so can sit any position on the chain. In a later version of the model [31,46] as also used in the current work, the slip-links hop discretely between neighboring monomers with acceptance rate controlled by a Metropolis Monte Carlo scheme. On average, one Monte Carlo hopping motion is attempted per slip-link per time step. The long time bebavior of the simulation system is not sensitive to the details of the slip-link hopping. Recently, Shivokhin et al. found that the slip-links can make non-negligible contributions to the effective friction experienced by the Rouse chain when moving along the tube because the virtual springs effectively restrict the excursion volumes of the slip-links and so reduce their successful rate to hop onto adjacent monomers [54]. Thus, an effective monomeric friction coefficient $\xi_{eff}$ should be used when mapping the simulation results of slip-spring model to experimental data. Such effect will not affect the discussion in the current study, since all data analysis and comparison are carried out within the same slip-spring model framework. The slip-spring model parameters are chosen to be $N_{e}^{ss}=4$ and $N_{s}^{ss}=0.5$ for consistence with previous publications [24,53,55,56].

Each arm is capped at the branch point such that the slip-links cannot slide through to other arms of the same star. The branch points are fixed in space instead of fluctuating around for the convenience of analyzing the slip-link motion along the arms. It has been shown by Masubuchi et al. in primitive chain network simulations that for asymmetric star polymers the simulations with branch points fixed in space predicted much slower stress relaxation than those allowing them to fluctuate [48]. A recent multichain slip-spring simulation study of Masubuchi also implies that fluctuations around the branch points may play a significant role in the stress relaxation of weakly entangled asymmetric stars or H-polymers [49]. For symmetric stars, the effect of branch point fluctuations is less significant because these branch points are essentially trapped in entanglement cages [49]. Our simulation work on melts of monodisperse symmetric star polymers has demonstrated that, even though the single-chain slip-spring model with fixed branched points predicted slower dynamics than MD simulations based on bead-spring model, the stress relaxation data obtained using the two different simulation methods show good qualitative agreement [31]. The branch point fluctuation effect can be further reduced in the non-CR systems due to the quenching of constraint release. As will be seen later in Section 6, our slip-spring simulation results on dielectric loss of symmetric stars agree reasonably well with experimental data obtained from star probes blended in long chain matrix. Therefore, at least for symmetric stars with arm lengths in the range we studied (4–8 entanglements), allowing the branch points to fluctuate will not change the slip-spring model predictions qualitatively from those obtained with fixed branch points. Some quantitative differences are however to be expected, especially in the terminal regime where a broader distribution of the innermost primitive path segments can be resulted from branch point fluctuations, which, in turn, can change the terminal relaxation times slightly. However, this will not alter the validity of the discussions and main conclusions drawn in this work.

Furthermore, the slip-links are not allowed to cross each other or sit on the same monomer. This assumption effectively introduces excluded volume interactions between the slip-links, which is supported by the low swapping rate between neighboring entanglements as found in the persistent close-contact analysis in our recent MD simulation study of star polymer melts [31]. We noticed that, in some of the recently developed multi-chain slip-spring models [36,37,38,39], the slip-links are allowed to cross over each other. There are additional short-range repulsions among all Rouse monomers have been introduced for maintaining homogeneous distributions of slip-links in space. In our single-chain slip-spring model, the probability distributions of the slip-links along the star arms have been shown to follow exactly those expected for one-dimensional real gas in equilibrium [46,57]. The excluded volume effects between slip-links is a key mechanism causing CR-induced drifting behaviors of entanglements towards the arm free ends [31].

During either creation or destruction of a slip-link (as our simulations are at time-reversible equilibrium), at least one arm free end would be involved. In the systems with constraint release, if a slip-link reaches the free end of the arm, it sits on and is then deleted from the system, its associated partner on another arm will also be deleted regardless of its location. At the same time, a new pair of coupled slip-links will be added to the system in the manner that one goes to the free end of a randomly chosen arm and the other is attached to a monomer of any other arm with an equal probability. The total number of slip-links is thus kept constant, which is reasonable for a system with large number of molecules at equilibrium. Since we will only concentrate on the arm-retraction mechanism in the current work, the CR mechanism is switched off by decoupling the association between the slip-links. The creation and destruction of slip-links can only take place at the free ends of the star arms. The system studied here is closely analogous to that addressed in Pearson–Helfand theory [9], where isolated star polymers are confined in a permanent network and so each entanglement is constructed by an arm strand and a network strand. Under such framework, the release of any entanglement can only be induced by arm retraction.

For each system we studied, an ensemble of hundreds of independently relaxing symmetric stars was simulated. The total number of slip-links in the system was determined by the number density $1/N_{e}^{ss}$ and kept constant throughout the simulation run. The initial configurations of the star arms were created by random walks with average step size $b_{3D}=1$. The slip-links were randomly allocated on monomers of different arms. The total simulation time highly depends on the arm length *N* because the terminal relaxation time $\tau_{d}$ of the star polymers grows exponentially with increasing *N* in the absence of CR. For the systems with N<40, the simulations ran for at least $50\tau_{d}$. For example, the terminal relaxation time of the stars with N=36 was found to be $\tau_{d}\approx 10^{6}$ from the arm end-to-end vector correlation function. The simulation of the corresponding system was run for a total time duration of $10^{8}$. The system was first equilibrated for a time interval of $\tau_{d}$ and the data were collected for analysis thereafter. For the systems with the longest arm length (N=42) we studied, the data were collected only over approximately one $\tau_{d}$ due to high computational cost. In all of the following analyses, the average locations of the slip-links refer to their mean positions averaged over their entire lifetimes.

The physical quantity in the limelight of this work is the mean first-passage time τ(s) of “slip-links” as a function of the fractional distance *s* away from the mean equilibrium position of the arm free ends, which is closely associated with the tube survival function μ(t). The development and validation of any quantitative theories for describing the dynamics of entangled branched polymers will rely on the availability of exact τ(s) data. For example, τ(s) can be used to test the validity of the arm retraction potential proposed in different theoretical models. It is important to note that, in the star polymer system without CR, the obtained tube survival function μ(t) represents the stress relaxation function G(t) of the system.

## 3. Constructing Primitive Paths in Slip-Spring Model

The confining tube as used in tube theories is a mean-field concept describing a tube-like region where the lateral motion of an entangled polymer is restricted to [2]. A clear microscopic definition of this region remains unsettled in real polymer systems, which causes difficulties for directly verifying the assumptions and predictions made in existing tube theories. Instead, it is more convenient to use the primitive path which represents the minima of the effective constraining potentials acting on the monomers of the target polymer. Two complementary views of the PP have been fruitfully investigated in theoretical calculations. In one view, the primitive path of a chain is treated as a continuous curvilinear path going through the center of the confining tube, which is a random walk with persistence length of the order of tube diameter *a*. In molecular dynamics simulations, such types of PPs have been constructed using the mean path (MP) [27] and isoconfigurational ensemble (ICE) [58] methods, which have no strong perturbation to the local structures of the polymer chains. In the second view, the primitive path can be constructed as a sequence of straight lines connecting consecutive entanglement points along a given chain. This idea has been implemented in the primitive path analysis (PPA) of MD trajectories of entangled polymer melts by shrinking the chains via total energy minimization or length minimization algorithms but not allowing them to cross each other. The disadvantage of PPA methods is that the chain-shrinking process destroys the local structures of the melts from their equilibrium configurations [19,59,60,61,62].

**Figure 1:**
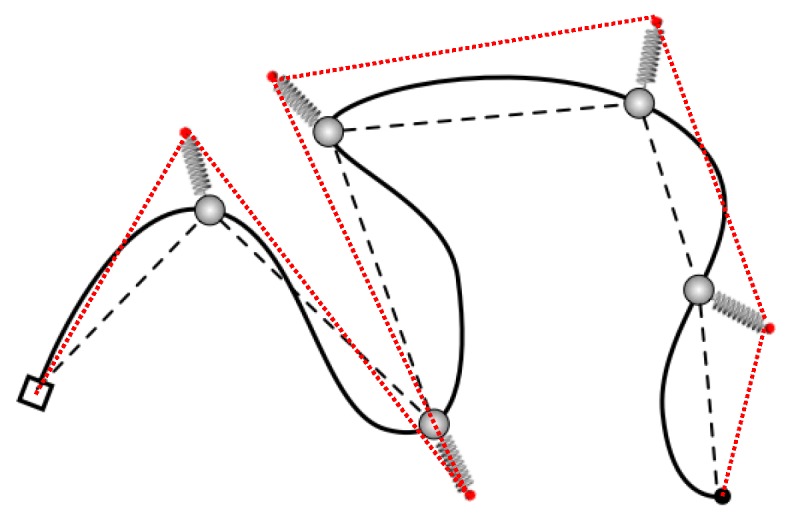
Sketch of a star arm simulated in the slip-spring model and the primitive paths constructed by connecting the successive anchor points (red dotted line) and the average locations of successive slip-links during their lifetimes (black dashed line), respectively.

The nature of the single-chain slip-spring model where the entanglements are introduced as discrete slip-springs or slip-links makes it remarkably simple to adopt the discrete picture of the primitive path. Different from PPA methods, there is no perturbation on the equilibrium structures of the system. The sketch of a single star arm at equilibrium is shown in Figure 1, in which the square and black disk symbols represent the branch point and arm free end, respectively. The slip-links (grey spheres) located on individual monomers of the arm are connected to anchor points fixed in space(small red spheres) through the virtual harmonic springs. The two alternative descriptions of the primitive path, namely by connecting either the successive anchor points or the average locations of successive slip-links during their lifetimes, are shown in Figure 1 by the red dotted and black dashed lines, respectively. Since we are also interested in recovering the arm end-to-end vector relaxation function ϕ(t), the segment between the arm free end and the outermost slip-link or anchor point is also included as part of the primitive path. The so-constructed PPs can be conveniently used for determining the tube model parameters through the static and dynamical properties of star polymers obtained in the slip-spring model simulations.

## 4. Static Properties: Primitive Path Analysis

**Figure 2:**
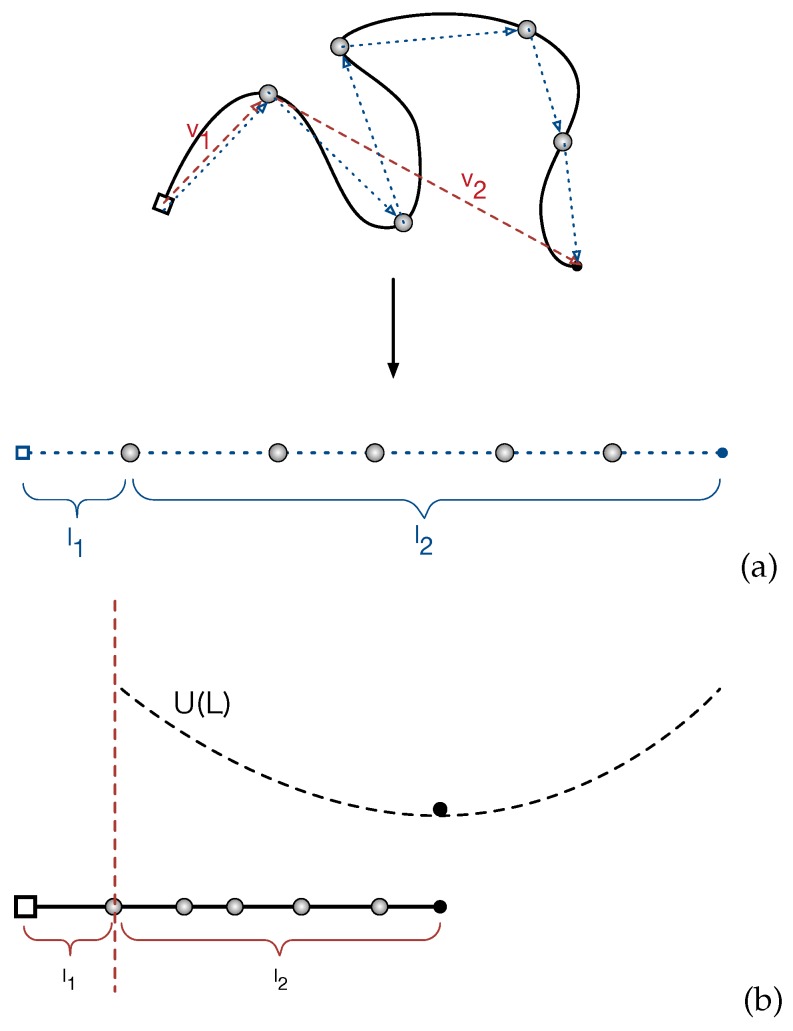
(**a**) Projection of the three-dimensional primitive path of a given star arm on a one-dimensional Rouse chain which preserves the tube segment lengths between the neighboring entanglement points (gray spheres). In the 3D plot of the arm, ${\vec{v}}_{1}$ is the vector pointing from the branch point to the inner-most entanglement point, and ${\vec{v}}_{2}$ is the vector from the inner-most entanglement point to the arm free end. In the 1D plot, $l_{1}$ corresponds the segment length of ${\vec{v}}_{1}$, while $l_{2}$ is the sum of the primitive path segment lengths between the inner-most entanglement point and the arm free end (not the length of ${\vec{v}}_{2}$); (**b**) mapping of the 1D Rouse chain obtained in (**a**) to a simplified theoretical model consisting of a single bead fluctuating under an effective arm retraction potential U(L), which has a minimum at the mean primitive path length 〈L〉.

We start with analyzing the distribution of the PP lengths of the entangled star arms in equilibrium state. In the discrete picture, the primitive path of a given star arm is a 3D random walk of *Z* steps with the average step length *a*. The corresponding PP length of the arm is then L=Za. Since the retraction of the arm free end proceeds in a curvilinear way along the PP, the 3D random walk can be projected on a 1D Rouse chain for convenience of theoretical calculations (see Figure 2a). As mentioned in Section 3 and sketched in Figure 1, the entanglement points (gray spheres) in the PP and the projected Rouse chain could be either the locations of the anchor points of the slip-springs or the mean positions of the slip-links on the given arm. In the setup of the single-chain slip-spring model, the initial configurations of the star arms are constructed as random walks with $Z=N/N_{e}^{ss}$ steps and step length equal to $\sqrt{N_{e}^{ss}}b$.

In the Pearson–Helfand theory, the 1D Rouse chain was further simplified to a coarse-grained bead, which is connected to the branch point through a harmonic spring and bears an entropic force $F_{ent}=\frac{3k_{B}T}{a}$ to preserve the average primitive length 〈L〉=Za, where $Z=N/N_{e}$ is the average number of entanglements per arm [9]. Under this assumption, the probability distribution of the PP length *L* at equilibrium is determined by the potential of the harmonic spring [2,3,63],

$$P\left(L\right)∼\exp\left(-\frac{U(L)}{k_{B}T}\right),$$

where the effective arm retraction potential is

(1) $$U\left(L\right)=\frac{3k_{B}TL^{2}}{2Nb_{3D}^{2}}-LF_{ent}.$$

The assumed potential form U(L) can be tested by using the probability distribution of the PP lengths obtained from our slip-spring model simulations.

Figure 2b sketches the mapping of the 1D Rouse chain representing the PP of a given arm in our slip-spring model system to the simplified theoretical model with a single bead fluctuating under the effective potential U(L). The potential is at its minimum when the length of the primitive path is at the mean value 〈L〉. Since there is no constraint release, the free end of a star arm retracts along the primitive path via thermal fluctuations and destroys the original entanglements sequentially from the outermost one towards the branch point. Once the arm end reaches an entanglement, the original primitive path or tube segment between this entanglement point and its inner-neighboring entanglement will be completely forgotten after a time scale around $\tau_{e}$. We find that the entanglement relaxation time $\tau_{e}$ is about the same order of the time unit of the current applied slip-spring model. Thus, we assume that the conformation of the tube segment will be immediately forgotten once the arm end reaches the outer slip-link confining the segment. This assumption will not affect our conclusions later in the paper. Based on this discrete picture of the PP, the arm retraction process is considered to be completed in our slip-spring simulations when the arm free end reaches the innermost original entanglement for the first time. The corresponding first-passage time is recorded as the terminal relaxation time $\tau_{d}$ of the arm. This differs from the definition of $\tau_{d}$ in the continuous tube theories that is the FP time required for the arm free end to retract continuously all the way back to the branch point [2,9]. In order to made direct examination of U(L) in Equation (1), we will focus on analyzing the distribution of the PP length in between the innermost entanglement and the arm free end, which is defined as $l_{2}$ in Figure 2b, but bearing in mind that the length of the entire PP is $L=l_{1}+l_{2}$ where $l_{1}$ is the segment length in between the branch point and the innermost entanglement.

**Figure 3:**
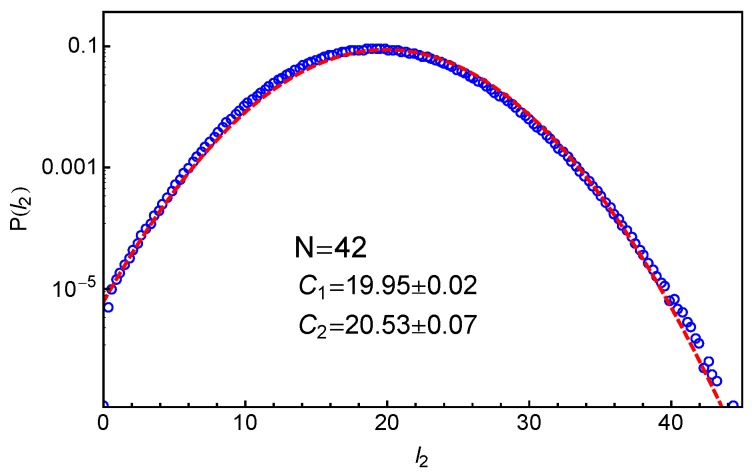
Probability distribution of the primitive path lengths $P(l_{2})$ in the system with star arm length N=42. Here, the primitive paths are defined using the locations of the anchor points of the slip-springs. The dashed line is the best fit to the Gaussian distribution in Equation (2).

Figure 3 shows our simulation results on the probability distribution of $l_{2}$ in the system with star arm length N=42, together with the best-fit to the Gaussian distribution

(2) $$P\left(l_{2}\right)=\frac{1}{\sqrt{2\pi C_{2}}}\exp\left(-\frac{{\left(l_{2}-C_{1}\right)}^{2}}{2C_{2}}\right),$$

where $C_{1}$ and $C_{2}$ are fitting parameters. Here, the primitive paths are determined using the locations of the anchor points of the slip-springs. It can be seen that the simulation data can be reasonably well described by the Gaussian distribution for the whole range of length scales and for $P(l_{2})$ over five orders of magnitude. This indicates that the effective arm retraction potential U(L) has a general quadratic form as assumed in Equation (1). The parameters $C_{1}$ and $C_{2}$ correspond to the mean value and variance of the PP length $l_{2}$, respectively, which are essential for extracting the tube model parameters as shown in the following sections.

**Figure 4:**
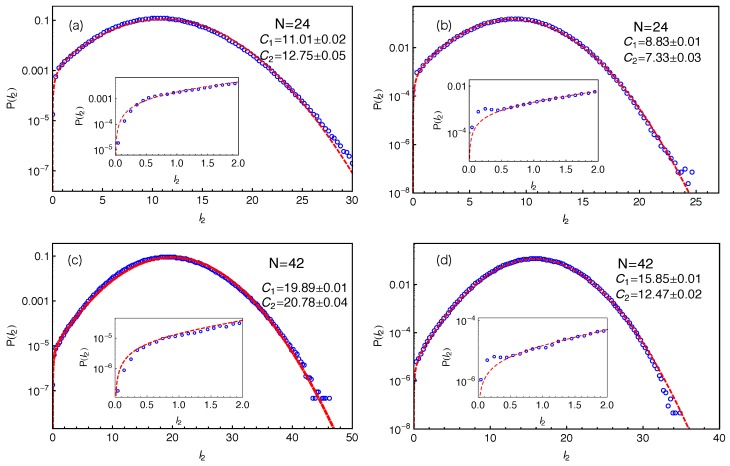
Probability distributions of primitive path lengths, $P(l_{2})$, of star arms with lengths N=24 (**a**,**b**) and 42 (**c**,**d**), respectively. In (**a**,**c**), the primitive paths are defined by connecting the successive anchor points, while in (**b**,**d**) by connecting the average locations of successive slip-links. The dashed lines are the best-fit of the simulation data to Equation (3). The insets show the $P(l_{2})$ data at very small $l_{2}$ values.

The distributions of tube lengths have also been studied by Likhtman and coworkers using simulations based on a simple grid model where the motion of a single Rouse chain is confined in a cubic lattice of line obstacles [64]. The tube length was measured in two different ways. One is the tube axis method by which the confining tube was constructed by connecting the centers of the cubes occupied by the given chain but removing all the cubes belonging to unentangled loops. The tube length was then calculated as $(Z_{c}-1)g,$ where $Z_{c}$ is number of cubes in the tube and *g* is the grid size. The other is the PP length which is the shortest distance between the chain ends obtained by fixing the chain ends and shrinking the chain by reducing the temperature without allowing it to cross the grid lines, analogous to the PPA method used in MD simulations [19,59,60,61,62]. The tube axis method is computationally cheaper and can produce data with better statistics. The obtained tube length distribution was found to deviate from the standard Gaussian form at improbable lengths L≪〈L〉 and L≫〈L〉. On the other hand, their results on the PP lengths are more scattered and so do not show significant deviation from the Gaussian distribution. For comparison, we present in Figure 4 our slip-spring model simulation results on the PP length $l_{2}$ for different arm lengths using two different PP constructions. In all cases, the results show good statistics even at very small $P(l_{2})$ values, which allow us to extract the tube theory parameters with high accuracy.

When fitting the simulation data on $P(l_{2})$ to the standard Gaussian function given in Equation (2), a truncation is found at the small $l_{2}$ side due to the fact that the PP length $l_{2}\geq 0$. Instead of employing a truncated Gaussian function with extra fitting parameters, we introduce a mirror image of the original Gaussian function such that the difference between these two functions is equal to zero at the origin as follows:

(3) $$\begin{array}{c} f\left(l_{2}\right)=\frac{1}{\sqrt{2\pi C_{2}}}\exp\left(-\frac{{\left(l_{2}-C_{1}\right)}^{2}}{2C_{2}}\right)-\frac{1}{\sqrt{2\pi C_{2}}}\exp\left(-\frac{{\left(l_{2}+C_{1}\right)}^{2}}{2C_{2}}\right). \end{array}$$

As shown in Figure 4, Equation (3) provides good descriptions of the simulation data for the entire range of $l_{2}$, in particular at small $l_{2}$ values—see the insets there. At larger PP lengths ($l_{2}\geq 2.5$), the fitting qualities of Equation (3) and the standard Gaussian form in Equation (2) to the $P(l_{2})$ data are nearly identical with very small adjustments in the fitting parameter values. This can be seen by comparing Figure 3 with Figure 4c and the $C_{1}$ and $C_{2}$ values given there. We notice in the insets of Figure 4b,d that the $P(l_{2})$ data show a small bump at $l_{2}\approx 0.2$, which probably results from the use of the time-averaged positions of the slip-links for constructing the PP, instead of their instantaneous positions. The existence of such small bump however will not affect our discussions below.

**Figure 5:**
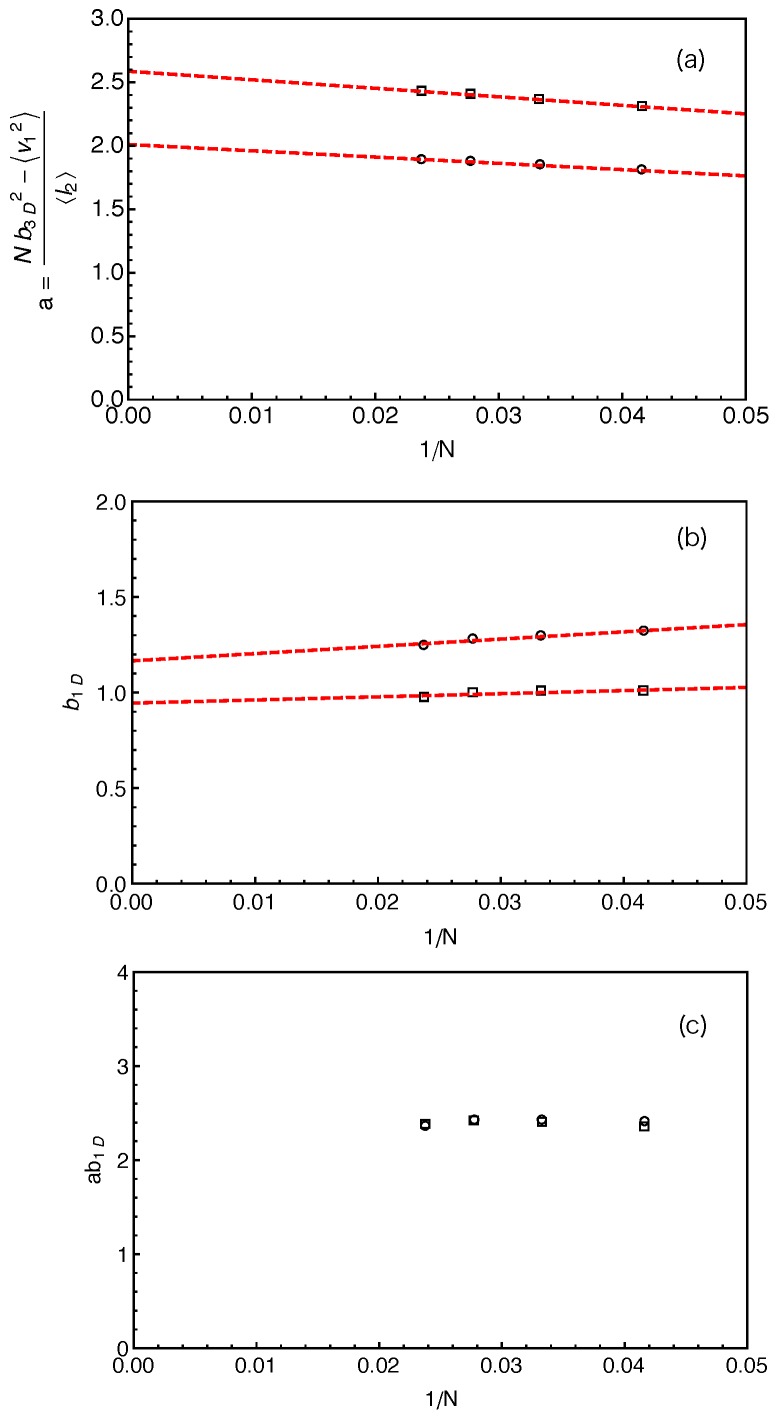
(**a**) Tube step length, *a*, obtained from Equation (4), (**b**) statistical segment length of 1D Rouse chain, $b_{1D}$, derived from Equation (6) and (**c**) the product $ab_{1D}$ as functions of the inverse arm length 1/N. The results are obtained by using two different constructions of the primitive path (circles for using anchor points and squares for using average positions of the slip-links).

We can now estimate the tube theory parameters using the results on PP lengths. Considering the random walk feature of the primitive path and the Rouse chain, we have the mean squared tube step length (or squared tube diameter) $a^{2}=N_{e}{b_{3D}}^{2}$ and the mean squared arm end-to-end distance $a\langle L\rangle =N{b_{3D}}^{2}$ where $b_{3D}$ is the statistical segment length of the chain in 3D space. Similarly, we have $a\langle l_{2}\rangle =\langle {\vec{v}}_{2}^{2}\rangle$ for the vector ${\vec{v}}_{2}$ pointing from the innermost entanglement point to the arm free end, as sketched in Figure 2. The average length of the primitive path in between these two points, $\langle l_{2}\rangle$, is given by the fitting parameter $C_{1}$ obtained from the best fit of Equation (3) to the simulation results on $P(l_{2})$. For a Rouse chain, the direction of ${\vec{v}}_{2}$ is spatially uncorrelated with that of the vector ${\vec{v}}_{1}$ which points from the branch point to the innermost entanglement point, which gives $N{b_{3D}}^{2}=\langle {\vec{v}}_{2}^{2}\rangle +\langle {\vec{v}}_{1}^{2}\rangle$. The mean tube step length or tube diameter *a* is then given by

(4) $$\begin{array}{c} a=\frac{\langle {\vec{v}}_{2}^{2}\rangle }{\langle l_{2}\rangle }=\frac{N{b_{3D}}^{2}-\langle {\vec{v}}_{1}^{2}\rangle }{\langle l_{2}\rangle }, \end{array}$$

where $\langle {\vec{v}}_{1}^{2}\rangle$ can be measured directly in our simulations based on the two different constructions of the primitive path as listed in Table 1. The *a* values calculated using Equation (4) are shown in Figure 5a as a function of inverse arm-length 1/N. The dashed lines are the linear fitting to the data sets. It can be seen that the estimated tube step length increases with the increase of the arm length in both cases. Extrapolating the data points to 1/N→0, we got the asymptotic tube step lengths in the long chain limit as a≈2.01 for the primitive path defined by anchor points and 2.59 for that defined by the average positive of the slip-links. The corresponding numbers of monomers per entanglement strand are then given by $N_{e}=a^{2}/{b_{3D}}^{2}\approx 4.0$ and 6.7, respectively. The *a* and $N_{e}$ values obtained by using the average positions of the slip-links are higher than those obtained using the anchor points, reflecting the longer PPs constructed by the former method for the same number of entanglements. In the single-chain slip-spring model, the instantaneous position of a slip-link fluctuates about the corresponding anchor point via the action of the virtual spring. If the slip-spring or entanglement survives infinitely long, the average position of the slip-link will converge to the location of the anchor point. However, due to arm retraction dynamics, the original slip-springs or entanglements along the arm are deleted by the arm free end in sequence. As a consequence, the average positions of the slip-links over their limited lifetimes deviate from the linked anchor points with certain randomness in the 3D space, leading to a longer PP winding around that constructed by the anchor points. In previous slip-spring model simulation works, a value of $N_{e}\approx 5.7$ was obtained by mapping the slip-spring model simulation results on the linear viscoelastic properties of linear polymer melts to the Likhtman–McLeish model predictions, [24,55] which sits in between the two asymptotic $N_{e}$ values estimated from the mean PP lengths $\langle l_{2}\rangle$. Likhtman et al. have shown recently that a so-called invariant number of segments between entanglements, $\tilde{N_{e}}$, can be defined based on the fluctuations of the PP lengths, which is much less sensitive to the details of the primitive path construction [64].

The fluctuations of the PP length around its mean value 〈L〉 can be characterized by the variance $\langle \Delta L^{2}\rangle =\langle {\left(L-\langle L\rangle \right)}^{2}\rangle$. Considering the 1D Rouse chain representing the curvilinear primitive path as sketched in Figure 2b, the variance of the chain end-to-end distance is independent of the stretching force at the arm free end and given by the Rouse theory as

(5) $$\begin{array}{c} \langle \Delta L^{2}\rangle =\frac{N{b_{1D}}^{2}}{3}, \end{array}$$

where the factor 3 is due to the fact that *L* is measured in one dimension only [64]. Here, $b_{1D}$ is the statistical segment length along the one-dimensional primitive path, which differs from the average bond length $b_{3D}$ of free Rouse chain in three dimensions, although $b_{1D}$ is generally assumed to be equal to $b_{3D}$ in the conventional tube theory. Since the PP segment lengths $l_{1}$ and $l_{2}$ are statistically independent from each other, we have $\langle \Delta L^{2}\rangle =\langle {\Delta l_{1}}^{2}\rangle +\langle {\Delta l_{2}}^{2}\rangle$. The variance of $l_{2}$ is nothing but $\langle {\Delta l_{2}}^{2}\rangle =C_{2}$ as obtained from the best-fit of the probability distributions $P(l_{2})$ to Equation (3). The variance of $l_{1}$ can also be conveniently measured in the simulations through $\langle {\Delta l_{1}}^{2}\rangle =\langle {{\vec{v}}_{1}}^{2}\rangle -\langle |{\vec{v}}_{1}{|\rangle }^{2}$. It follows from Equation (5) that

(6) $$\begin{array}{c} {b_{1D}}^{2}=\frac{3\left(\langle {{\vec{v}}_{1}}^{2}\rangle -\langle |{\vec{v}}_{1}{|\rangle }^{2}+\langle {\Delta l_{2}}^{2}\rangle \right)}{N}. \end{array}$$

The $b_{1D}$ results obtained by using two different primitive path constructions are shown in Figure 5b as a function of inverse arm length. The extrapolated asymptotic values of $b_{1D}$ in the long arm limit are listed in Table 1, which are not significantly departed from $b_{3D}=1$ in both cases.

The analytical calculations of Likhtman et al. suggested that, around the Rouse time $\tau_{R}$ the mean squared displacement, $g_{1,mid}\left(t\right)$, of the middle monomer of a confined Rouse chain in real space only depends on two combined parameters: $ab_{1D}N^{1/2}$ and $\tau_{R}$ [64]. It implies that, even though the individual *a* and $b_{1D}$ values may vary with the methods used for constructing the PPs, their product $ab_{1D}$ should be independent of the PP construction. This is confidently verified in our simulations as shown in Figure 5c for different arm lengths. One can then write the relative magnitude of the PP length fluctuations in terms of $ab_{1D}$ [64]

(7) $$\begin{array}{c} \frac{\langle \Delta L^{2}\rangle }{{\langle L\rangle }^{2}}=\frac{N{b_{1D}}^{2}}{3{\left(Za\right)}^{2}}=\frac{a^{2}b_{1D}^{2}}{3Nb_{3D}^{4}}=\frac{1}{3Z}, \end{array}$$

where we have used Equation (5), the mean PP length 〈L〉=Za and the mean squared end-to-end distance of the primitive path in 3D space $Za^{2}=Nb_{3D}^{2}$. In the last step of Equation (7), the average number of entanglements per chain is defined by $Z=N/\tilde{N_{e}}$ with the invariant number of monomers per entanglement

(8) $$\begin{array}{c} \tilde{N_{e}}\equiv \frac{a^{2}b_{1D}^{2}}{{b_{3D}}^{4}}. \end{array}$$

Since $b_{3D}$ and $ab_{1D}$ are either independent or insensitive to the details of the PP construction, the $\tilde{N_{e}}$ values should also be insensitive to the different PP constructions we used. This is again confirmed by our simulation results in Table 1 where the two $\tilde{N_{e}}$ values are shown to be very close to each other and both are in good agreement with that ($N_{e}\approx 5.7$) obtained from mapping slip-spring simulation results on linear viscoelastic properties to tube model predictions [24,55]. Therefore, our primitive path analysis provides strong evidence to support the suggestion of Likhtman et al. [64] that the use of the first two moments of the PP length distribution, 〈L〉 and $\langle L^{2}\rangle$, rather than only 〈L〉, is essential to extract the key tube theory parameter $N_{e}$ without significant dependence on the details of the primitive path construction. It should be noted that if $b_{1D}=b_{3D}\left(=b\right)$, Equation (8) reduces the standard tube theory definition $\tilde{N_{e}}=N_{e}=a^{2}/b^{2}$. In the long chain limit, the star polymer systems we studied are close to that situation as shown by the simulation results on $b_{1D}$ in Figure 5b and Table 1. In the following, we will use the value of $\tilde{N_{e}}\approx N_{e}\approx 5.7$ to evaluate the number of entanglements per arm, *Z*.

## 5. Dynamic Properties: First-Passage Time of Entanglement Disengagement

As mentioned in the Introduction, dynamics of arm retraction in a fixed polymer network can be formulated as a first-passage time problem and has been solved by various theoretical models [9,11,14]. In this section, we will examine the predictions of these theoretical works and extract the related theory parameters by studying the FP times of entanglement disengagement along the confined star arms. Since entanglements are represented by discrete slip-links in the slip-spring model, we first introduce a convenient and efficient method for measuring the distribution of the FP times of slip-link destruction.

Consider a star arm confined in a fixed polymer network where the monomer index increases from i=0 at the branch point to *N* at the arm free end. The primitive path of the arm can be constructed by the two methods sketched in Figure 1. A 1D coordinate *x* along the primitive path is defined as the distance measured from the branch point. Accordingly, the fractional retraction distance along the PP is defined as s=1−x/〈L〉, which is equal to 0 at the mean equilibrium position of the arm free end and 1 at the branch point [9,11,42,65]. Suppose that, at a reference initial time *t*, a target slip-link sits on monomer *i* and is a distance *x* away from the branch point along the PP. If this slip-link is destructed by the retracting arm end at a later time $t^{\prime }$, the FP time for disengaging an entanglement that is a PP distance *x* away from the branch point is then recorded as $\tau \left(x\right)=t^{\prime }-t$. Following the same procedure, the distribution of τ(x) at a given *x*, denoted as f(x,τ), can be obtained by performing a large set of slip-spring simulations and collecting the τ(x) data by tracking all the slip-links, which, at some time points during their lifetimes, have been located at the PP coordinate *x*. Such calculations, however, demand the storage of entire trajectories of the simulated systems for post-simulation data processing because the destruction times $t^{\prime }$ of the slip-links are not known in advance. This problem can be resolved by taking advantage of the single-chain slip-spring model and the *time reversible* feature of the equilibrium systems.

In the single-chain slip-spring model without constraint release, the slip-links can only be created and destructed by the arm free ends. Once a slip-link is created, its PP coordinate *x* will not change for the entire lifetime. Therefore, the reference initial time *t* for calculating the FP time can be chosen as any time point during the lifetime of this slip-link. Instead of waiting for the moment of its destruction, we refer to the fact that arm extension is essentially the reverse process of arm retraction. In other words, the dynamic process experienced by the slip-link from its creation to destruction should be statistically equivalent to the time reversible process for it to go from the moment of destruction to creation. It means that the same distribution of τ(x) at a given *x* can be obtained by either measuring the difference between any reference initial time during the lifetime of the target slip-link and its destruction time or between its creation time and any later time during its lifetime. By using the latter approach, we can collect the statistics of the FP times on the fly in simulations without saving the system trajectories. In more technical details, the creation time $t_{create}$ of each slip-link is recorded during a simulation run. The difference between the current time and $t_{create}$ of that slip-link, $t-t_{create}$, is counted as one FP time τ(x) for establishing the two-variable distribution function f(x,τ).

**Figure 6:**
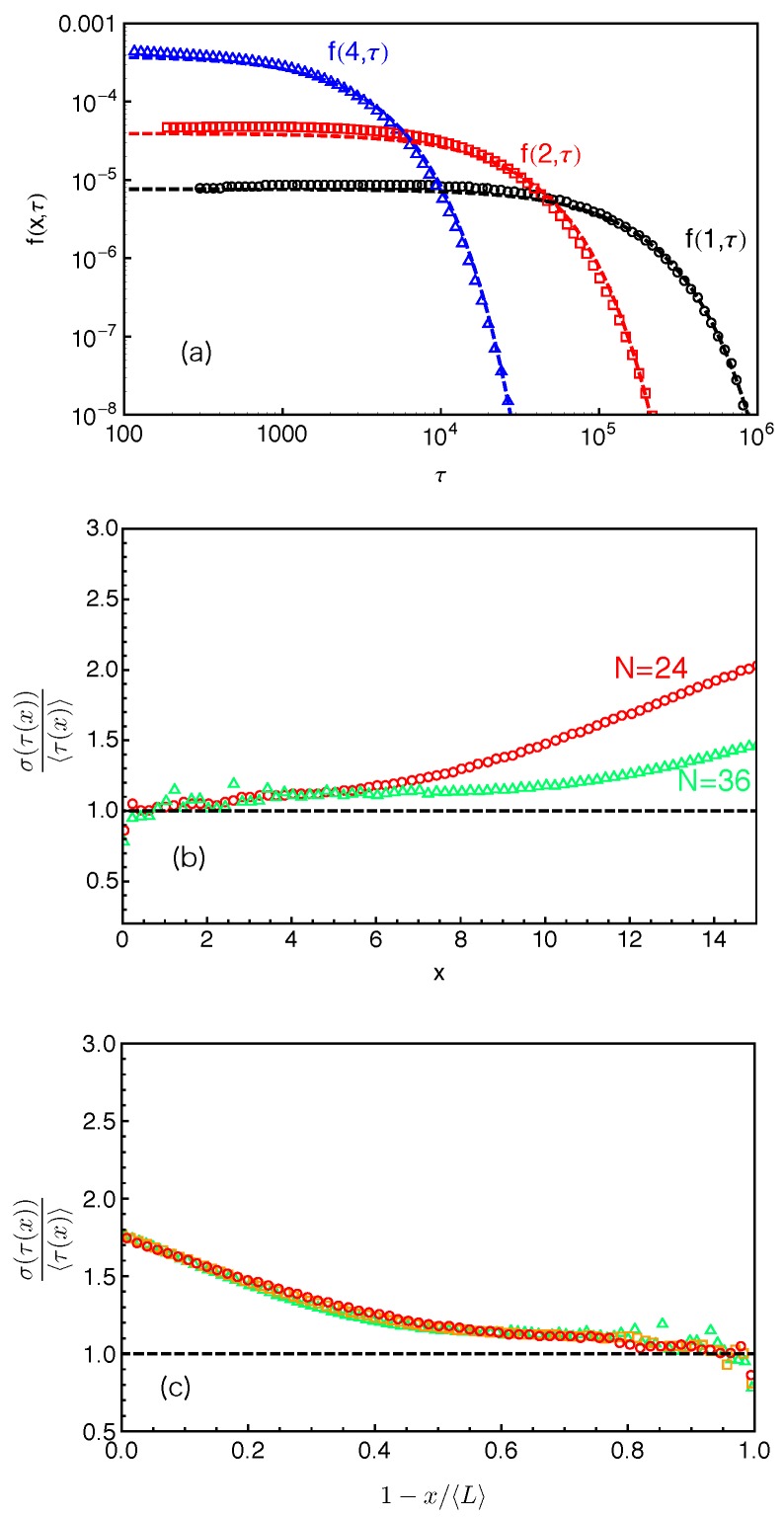
(**a**) Distributions of first-passage times f(x,τ) for destructing slip-links at different primitive path coordinates *x* in the system with arm length N=24. The dashed lines are best-fits of the simulation data (symbols) to the exponential function $\frac{1}{\tau_{c}}\exp\left(-\tau /\tau_{c}\right)$, where the characteristic times are found to be $\tau_{c}=2.6\times 10^{3}$, $2.5\times 10^{4}$ and $1.3\times 10^{5}$ for x=4, 2 and 1, respectively; (**b**) ratio between the standard deviation $\sigma \left(\tau (x)\right)$ and the mean 〈τ(x)〉 of the FP time distribution as a function of the primitive path coordinate *x* in the systems with N=24 and 36; (**c**) the same $\frac{\sigma (\tau (x))}{<\tau (x)>}$ data as in (**b**) as a function of the fractional distance along the primitive path s=1−x/〈L〉 for the systems with N=24 (circles), 30 (squares) and 36 (triangles), respectively.

Figure 6a presents the distributions of the FP times f(x,τ) for destructing slip-links at different PP distances (x=1,2 and 4 based on anchor points) away from the branch points in the system with arm length N=24 (Z≈4). The dashed lines there represent the best-fits of the simulation data to the exponential function $\frac{1}{\tau_{c}}\exp\left(-\tau /\tau_{c}\right),$ where $\tau_{c}$ is the characteristic time of the distribution at a given *x*. Our simulation results clearly show that, for the slip-links or entanglements close to the branch points, the FP time distributions of their destruction generally follow the exponential form. The characteristic time $\tau_{c}$ increases dramatically with the decrease of *x* due to the higher entropic energy barrier that the arm free end needs to overcome for reaching the corresponding slip-links.

The mean value of an exponentially distributed variable is equal to its standard deviation. We can thus use the ratio between the standard deviation $\sigma \left(\tau (x)\right)$ and the mean 〈τ(x)〉 of the FP time τ(x) to quantify how well the distributions f(x,τ) at different *x* values follow the exponential form. Simulation results on $\sigma \left(\tau (x)\right)/\langle \tau \left(x\right)\rangle$ are given in Figure 6b for the systems with arm lengths N=24 and 36. In the system with N=24, the ratio is almost exactly (at x≤2) or only slightly above 1 up to x≈5, which is consistent with the good fitting qualify in Figure 6a. The data points in the system with N=36 (Z≈6.3) are rather scattered at small *x* values. The relatively poor statistics arises from the long terminal arm relaxation time in the system (about $10^{7}\tau_{0}$), which is not sufficiently sampled in the standard slip-spring model simulations with limited computational power. More accurate results can be achieved by combining the single-chain slip-spring model with forward flux sampling method [14,46], but this will not affect our physical discussions in the current work. Figure 6b shows that $\sigma \left(\tau (x)\right)/\langle \tau \left(x\right)\rangle$ obtained at N=36 stays close to 1 up to x≈8 in the longer arm systems. The PP coordinate at which the f(x,τ) data deviate from the exponential distribution thus shifts to higher *x* values with the increase of the arm and so mean PP lengths. It implies a possible universal behavior of the FP time distributions as a function of the relative distance $\frac{x}{\langle L\rangle }$ from the branch point or the fractional retraction distance $s=1-\frac{x}{\langle L\rangle }$ along the PP.

Figure 6c plots the $\sigma \left(\tau (x)\right)/\langle \tau \left(x\right)\rangle$ data as a function of the fractional retraction distance *s* for the systems with arm lengths N=24, 30 and 36. All three data sets fall reasonably well onto a universal curve, indicating that the FP time distributions f(s,τ) follow the exponential form if the slip-links or entanglements are close to the branch points, but become non-exponential for the entanglements at small fractional distances *s* from the mean equilibrium positions of the arm free ends. In the latter case, the arm free ends undergo Rouse-like fluctuations around their mean equilibrium positions. Tube theories based on assuming an arm retraction process over high energy barrier do not apply to describing such shallow or early time fluctuations where the barrier height is less or comparable to $k_{B}T$. Using the arm retraction potential $U\left(s\right)=\nu Zs^{2}$ where the prefactor ν is 3/2 in the PH model [9] and 15/8 in the MM model without CR [11], the transition from early-time non-exponential to late-time exponential behavior is theoretically predicted to take place around $s\approx {\left(\nu Z\right)}^{-1/2}$. For the arm lengths $4.2<Z(=N/\tilde{N_{e}})<6.3$ studied in Figure 6c, this corresponds to a transition region of 0.60<s<0.68 for ν=3/2 or 0.64<s<0.71 for ν=15/8. The simulation data on $\sigma \left(\tau (x)\right)/\langle \tau \left(x\right)\rangle$ show a clear trend to approach value 1 in the predicted *s* region, taking into account the uncertainty in the ν and $\tilde{N_{e}}$ values. This also elucidates the applicability of the quadratic form of U(s) for describing the deep arm retraction dynamics, which is consistent with its validity in describing the static properties as discussed in Section 4.

**Figure 7:**
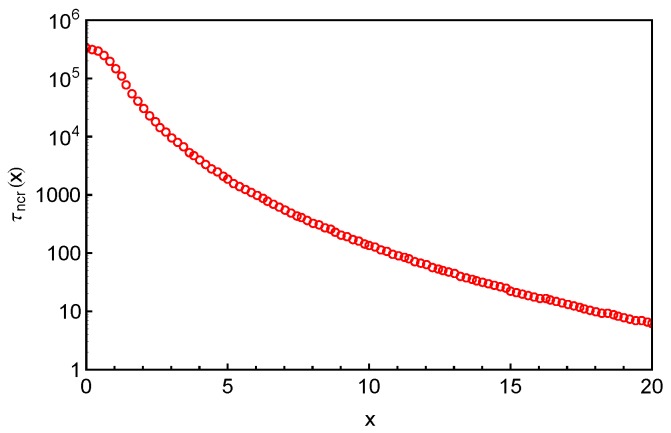
Mean first-passage time $\tau_{ncr}\left(x\right)$ of slip-links at primitive path coordinate *x* in the system with arm length N=24.

**Figure 8:**
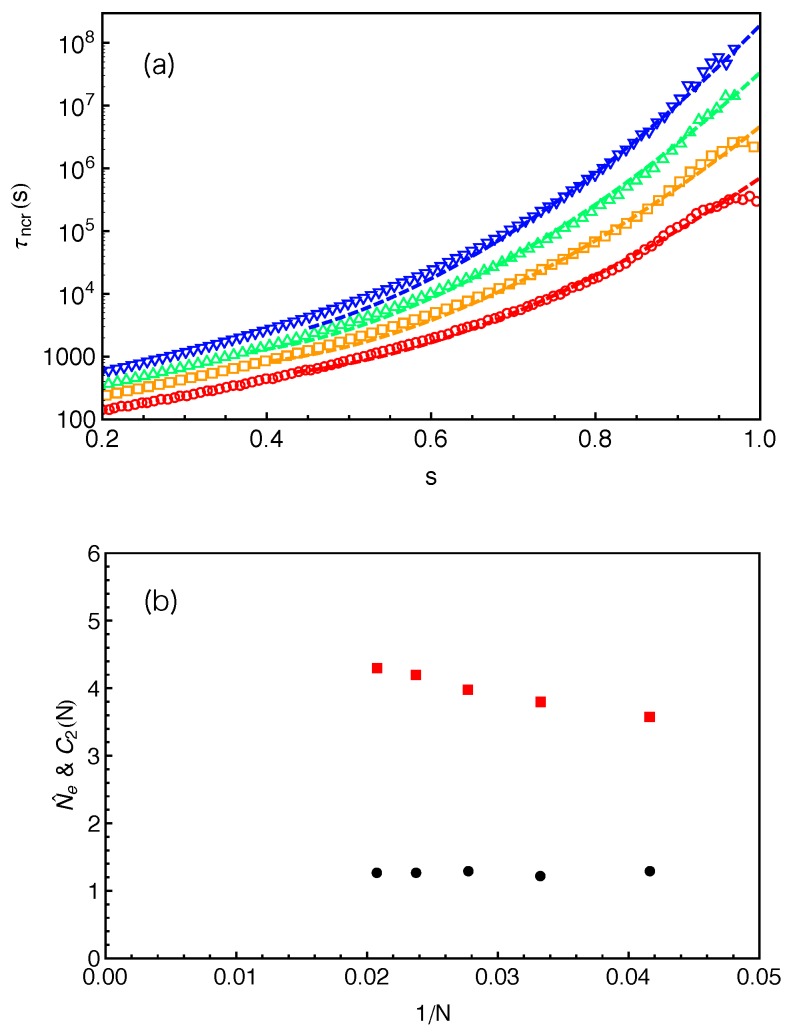
(**a**) Mean first-passage time $\tau_{ncr}\left(s\right)$ as a function of the fractional retraction distance *s* for the systems with different arm lengths N=24,30,36 and 42 from bottom to top. The dashed lines are the predictions of the large deviation theory (Equation (10)) made using the fitting parameters $C_{2}\left(N\right)$ (black disks) and $\tilde{N_{e}}$ (red squares) given in (**b**).

The mean FP time $\tau_{ncr}\left(x\right)\left(\equiv \langle \tau \left(x\right)\rangle \right)$ of slip-links destructed at PP coordinate *x* can be calculated using the distribution f(x,τ) obtained in simulations

(9) $$\begin{array}{c} \tau_{ncr}\left(x\right)=\int_{0}^{+\infty }\tau f\left(x,\tau \right)d\tau , \end{array}$$

where the subscript ncr indicates that there is no CR effect in the system. The $\tau_{ncr}\left(x\right)$ results for the system with N=24 are presented in Figure 7 by a linear-log plot over a *x* range beyond the average PP length 〈L〉≈12.57 of the studied arms. Apart from the terminal regime at very small *x*, the FP time grows exponentially with the arm retraction depth, which qualitatively agrees with the prediction of the PH theory [9]. The terminal arm relaxation time in the system is found to be $\tau_{d}\approx 3\times 10^{5}\tau_{0}$. The same FP data have also been plotted in Figure 8a as a function of the fractional retraction distance *s*, together with the results obtained from systems with arm lengths N=30,36 and 42. As expected, the $\tau_{ncr}\left(s\right)$ curves shift to higher time scales with the increase of the arm length. For the systems with longer arms (N≥36), the exponentially slow dynamics makes it difficult in simulations to well sampling the terminal relaxation regime. Therefore, the statistics of the FP times of the innermost slip-links and correspondingly the arm terminal relaxation times are not very satisfactory. However, the overall quality of the $\tau_{ncr}\left(s\right)$ curves are sufficient for being used to extract tube theory parameters.

In a previous work [14], we developed a large deviation theory of 1D Rouse chain with one end fixed for determining the mean FP time of the arm free end to reach certain improbable extension state. Here, we briefly describe the theory. As sketched in Figure 2b, the 3D primitive path of an entangled star arm in a fixed network can be projected onto a 1D Rouse chain with one end fixed at the origin. The problem of finding the tube survival probability is then simplified to solving a FP problem for the Rouse chain. The equations of motion of the Rouse beads can be transferred to a set of independent equations for the eigenmodes of the system, called Rouse modes. Thus, the original problem is transformed to a multi-dimensional Kramers’ problem. Such transition path problems are treated with the well-known Freidlin–Wentzell theory [66], which provides the statistical weight of each path in terms of an action functional and suggests that the most probable path is the one minimizing the action functional associated with the system. Following that, the multi-dimensional FP problem is further reduced to the conventional Kramers’ problem along the Minimal Action Path (MAP) that has an exact analytical solution for the mean FP time of a diffusing particle experiencing certain effective potential. In addition, we introduce an entropic correction to the effective free energy along the MAP by considering the fluctuations of the particle in a channel perpendicular to the MAP. Our theory predicts two scaling regimes of the mean FP time that the arm free end reaches a certain distance *s* away from its equilibrium position, namely $\tau \left(s\right)∼\frac{1}{s^{3}}\exp\left(U\left(s\right)/k_{B}T\right)$ in the intermediate *s* regime before the chain reaches its fully extension and $\tau \left(s\right)∼\frac{1}{s}\exp\left(U\left(s\right)/k_{B}T\right)$ at large *s* values. An empirical expression is then proposed to cover both regimes

(10) $$\begin{array}{c} \tau \left(s\right)=(\frac{C_{1}\left(N\right)}{N{\left(N/{\overset{ˇ}{N}}_{e}\right)}^{1/2}s}+\frac{C_{2}\left(N\right)}{{\left(N/{\overset{ˇ}{N}}_{e}\right)}^{3/2}s^{3}})\tau_{R}\exp(\frac{3N}{2{\overset{ˇ}{N}}_{e}}s^{2}), \end{array}$$

where the Rouse time $\tau_{R}=\frac{\xi b^{2}}{12k_{B}T}\sin^{-2}\left(\frac{\pi }{4(N+1/2)}\right)$, and $C_{1}\left(N\right)=\sqrt{\frac{32\pi }{3}}N^{2}\sin^{2}\left(\frac{\pi }{4(N+1/2)}\right)$. $C_{2}\left(N\right)$ and ${\overset{ˇ}{N}}_{e}$ are two fitting parameters. We note that this expression is applicable to the intermediate and large *s* regimes due to the use of the quadratic form of the arm retraction or extension potential.

**Figure 9:**
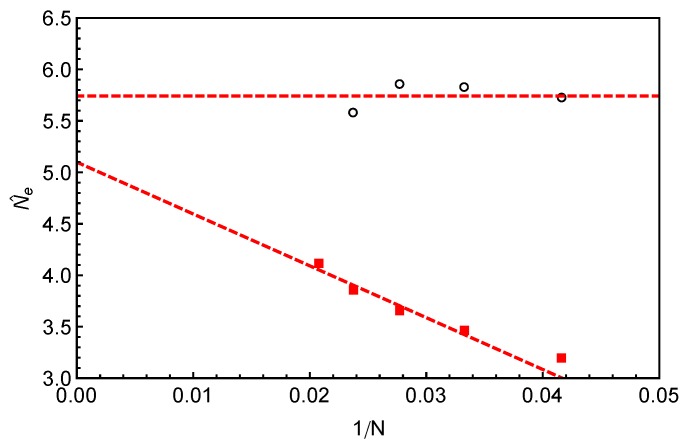
Numbers of monomers per entanglement obtained from static primitive path analysis using Equation (8) ($\tilde{N_{e}}$, cirlces) and dynamic first-passage time spectrum analysis using Equation (10) ($\overset{ˇ}{N_{e}}$, squares) for the systems with different arm lengths.

In Figure 8a, the predictions of Equation (10) made by using the fitting parameters $C_{2}\left(N\right)$ and ${\overset{ˇ}{N}}_{e}$ given in Figure 8b are compared with the slip-spring model simulation data for different arm lengths. For each *N*, reasonably good agreement between the theoretical and simulation data has been reached for the range of *s* covered by the theory. It is noticed that the $C_{2}$ values used for the fitting are around 1.2 for all the arm lengths studied, which is very close to that obtained from fitting Equation (10) to simulation data on τ(s) of 1D Rouse chain extension (instead of retraction) in Reference [14]. Therefore, we carry out another round of fitting by fixing $C_{2}\left(N\right)\;=\;1.2$ and using the number of monomers per entanglement strand ${\overset{ˇ}{N}}_{e}$ as the only fitting parameter. The obtained ${\overset{ˇ}{N}}_{e}$ values (red squares) are plotted in Figure 9 against 1/N, together with the invariant number of monomers per entanglement ${\tilde{N}}_{e}$ estimated using Equation (8) from the primitive path analysis. Different from ${\tilde{N}}_{e}$, the ${\overset{ˇ}{N}}_{e}$ values show a strong arm length dependence by growing roughly linearly with the inverse of *N*. At finite arm lengths, the $N_{e}$ values estimated from static (${\tilde{N}}_{e}$) and dynamic (${\overset{ˇ}{N}}_{e}$) analyses differ significantly from each other, e.g., by 40% for longest arm length N=42 studied in this work. In the long chain limit, the asymptotic value of ${\overset{ˇ}{N}}_{e}\approx 5.1$ is close but still smaller than the invariant value ${\tilde{N}}_{e}\approx 5.7$ by about 10%.

The large discrepancy between ${\tilde{N}}_{e}$ and ${\overset{ˇ}{N}}_{e}$ for a given arm length may be related to the different primitive path portions used for the static and dynamic analyses. The primitive path analysis in Section 4 was focused on the PP length $l_{2}$ in between the innermost slip-link and the arm free end because the arm retraction process is terminated in the discrete slip-spring model when the innermost slip-links are destructed. However, when calculating the FP time distribution f(x,τ), *x* is measured as the distance away from the branch point along the primitive path, which includes $l_{1}$ as sketched in Figure 2. Consequently, the FP times of the innermost slip-links are collected and counted into the distribution function f(x,τ) over a certain range of x≥0 or equivalently $l_{1}\geq 0$, depending on their locations on the PPs. As will be seen in Section 6, the f(x,τ) functions calculated this way are required for providing tube survival probabilities μ(t) consistent with the standard tube theory definition. If we instead define *x* as the distance measured from the innermost slip-link along the PP, the FP times of destructing all innermost slip-links will be counted to a single point (x=0) in the probability distribution, i.e., f(x=0,τ). This introduces a discontinuity in the FP time spectrum $\tau_{ncr}\left(s\right)$ at the corresponding fractional distance $s=1-x/\langle l_{2}\rangle =1$. The resulted f(x,τ) distributions also do not follow the exponential form even at small *x* values (not shown).

As discussed in the previous sections, the terminal relaxation time of the arm $\tau_{d}$ is determined by the mean first-passage time of the free arm end reaching the innermost slip-link. Thus, after transferring from absolute length scale *x* to the fractional distance *s*, $\tau_{d}$ would normally contribute to $\tau (s\approx \frac{Z-1}{Z})$ instead of τ(s≈1). As an attempt to make the definitions of the primitive path and fractional distance *s* used in the FP time analysis more close to those used in the primitive path analysis, we introduce an ad hoc renormalization of the fractional distance $s^{\prime }=\frac{Zs}{Z-1}=s\left(1+\frac{\overset{ˇ}{N_{e}}}{N-\overset{ˇ}{N_{e}}}\right)$ so that the FP times of the innermost slip-links would contribute to $\tau_{ncr}\left(s^{\prime }\approx 1\right)$ instead of $\tau_{ncr}\left(s\approx \frac{Z-1}{Z}\right)$. The modified expression of the mean FP time of arm-retraction is then given as

(11) $$\begin{array}{c} \tau \left(s\right)=(\frac{C_{1}\left(N\right)}{N{\left(N/{\overset{ˇ}{N}}_{e}\right)}^{1/2}s\left(1+\frac{\overset{ˇ}{N_{e}}}{N-\overset{ˇ}{N_{e}}}\right)}+\frac{C_{2}\left(N\right)}{{\left(N/{\overset{ˇ}{N}}_{e}\right)}^{3/2}\left(s(1+\frac{\overset{ˇ}{N_{e}}}{N-\overset{ˇ}{N_{e}}})\right){}^{3}})\tau_{R}\exp(\frac{3N}{2{\overset{ˇ}{N}}_{e}}\left(s(1+\frac{\overset{ˇ}{N_{e}}}{N-\overset{ˇ}{N_{e}}})\right){}^{2}). \end{array}$$

**Figure 10:**
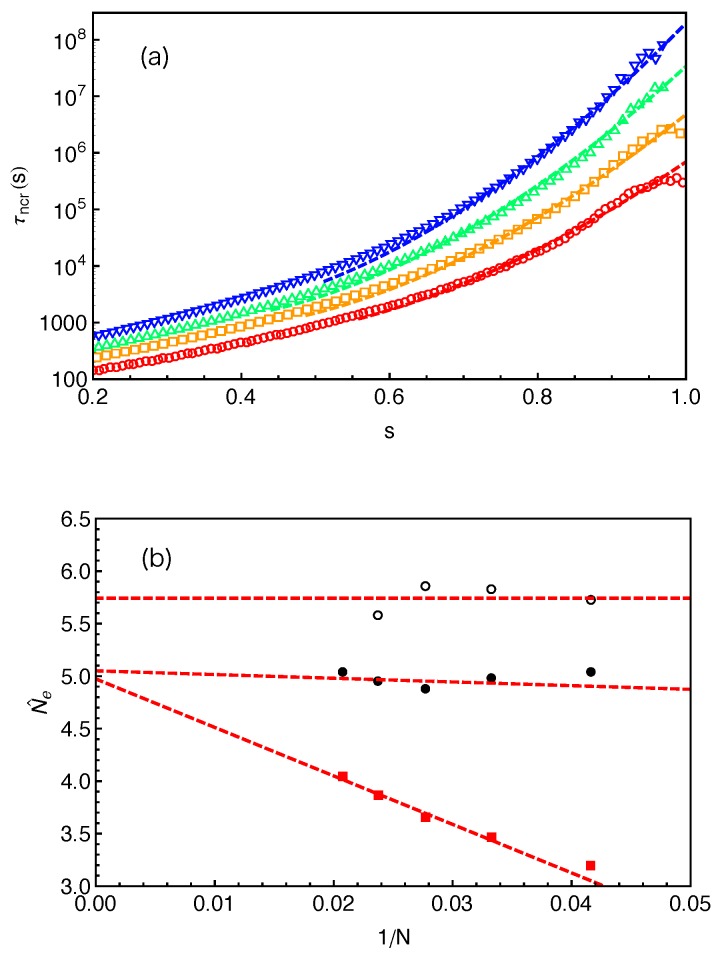
(**a**) Mean first-passage time spectra $\tau_{ncr}\left(s\right)$ for the systems with different arm lengths (symbols, same as in Figure 8). The dashed lines are predictions made by using the large deviation theory (Equation (11)) with renormalized fractional distance $s^{\prime }$ and the fitting parameters ${\overset{ˇ}{N}}_{e}$ (black disks) given in (**b**). The $\tilde{N_{e}}$ and ${\overset{ˇ}{N}}_{e}$ results from Figure 9 are also included in (**b**) for comparison.

Equation (11) will reduce to Equation (10) in the long chain limit as $s^{\prime }\to s$. We then fit Equation (11) to the slip-spring simulation data on τ(s) following the same fitting procedure as done with Equation (10), namely using $C_{2}\left(N\right)=1.2$ and $\overset{ˇ}{N_{e}}$ as the only fitting parameter. The fitting curves are shown as dashed lines in Figure 10a and the fitting parameters $\overset{ˇ}{N_{e}}$ are given in Figure 10b (black disks) together with the $\tilde{N_{e}}$ and ${\overset{ˇ}{N}}_{e}$ results from Figure 9. The fitting quality of Equation (11) to the τ(s) data in the relevant *s* region is similar to that of Equation (10), but the fitting parameters $\overset{ˇ}{N_{e}}$ show very different *N* dependence in these two cases. The ones obtained using Equation (11) remain nearly constant, independent of the arm lengths we studied. Nevertheless, the asymptotic $\overset{ˇ}{N_{e}}$ values are very close but both are smaller than the invariant value of ${\tilde{N}}_{e}\approx 5.7$.

The large deviation theory has also been used to describe the simulation results on the terminal relaxation times $\tau_{d}\left(N\right)$ of star arms in the absence CR obtained by using a combined slip-spring and forward flux sampling method [46]. There the arm length studied up to N=72 and $\tau_{d}$ was defined as the FP time for destructing the innermost slip-link. The $\tau_{d}\left(N\right)$ data were fitted with the theoretical prediction of $\tau_{d}\left(N\right)=C_{2}\left(N\right)\tau_{R}\left(N\right)\exp(3N/2{\overset{ˇ}{N}}_{e})/(N/{\overset{ˇ}{N}}_{e}){}^{3/2}$ with $C_{2}\left(N\right)=1.2$. The obtained ${\overset{ˇ}{N}}_{e}$ values also showed a linear increase with 1/N, qualitatively similar to that in Figure 9. However, the asymptotic ${\overset{ˇ}{N}}_{e}$ value of about 4.47 found in that work is lower than ${\overset{ˇ}{N}}_{e}\approx 5.1$ given in Figure 9. On the other hand, the fitting of the same $\tau_{d}\left(N\right)$ data with the Milner–McLeish theory without CR yielded a nearly constant value of $N_{e}\left(N\right)\approx 4.94$ [46]. The systematically lower $N_{e}$ values found in the previous work may be attributed to the neglect of the relaxation of the last tube or primitive path segment in between the innermost slip-link and branch point by defining $\tau_{d}$ as the FP time for destructing the innermost slip-link. Neglecting the relaxation process of the innermost PP segment has a more noticeable effect on determining the exact terminal relaxation time than on the overall shape of the FP time spectrum τ(s) when comparing with theoretical predictions based on continuous tube assumption. Since this is a common problem for all discrete simulation models, we will propose an analytical approach to take into account the innermost PP segment relaxation for constructing continuous relaxation functions using the FP time spectrum obtained from discrete model simulations.

## 6. Determining Relaxation Functions Using First-Passage Times of Entanglement Disengagement

As discussed above, FP times of entanglement disengagements play an essential role in determining the dynamics of entangled polymers. According to tube theories, they can be applied to calculate the tube survival probabilities and consequently the important time correlation functions, such as the stress relaxation function G(t) and the end-to-end vector correlation function of polymer chains ϕ(t), which are directly measurable in experiments [2,9,11]. In the absence of constraint release, all entanglements sitting on a star arm are released by the arm free end. Therefore, both G(t) and ϕ(t) are proportional to the survival probabilities of all the original entanglements or tube segments [2]. For star polymer melts with CR, it has been shown by MD and slip-spring simulations that G(t) is determined by the survival probabilities of all entanglements in the system, while ϕ(t) of a target arm is dominated only by the entanglements destroyed from the arm free end [31].

**Figure 11:**
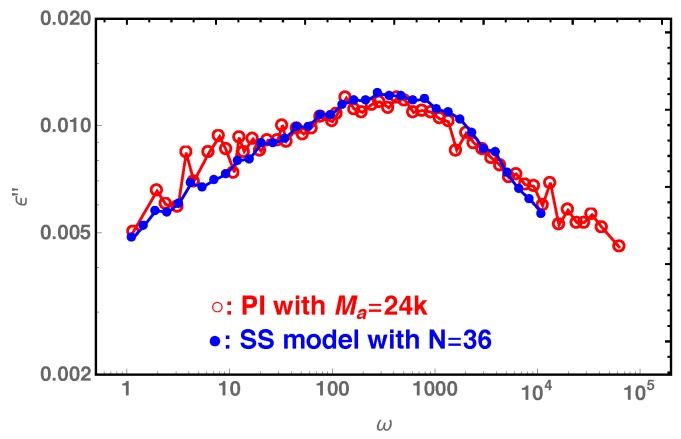
Comparison of dielectric loss spectra $\epsilon^{\prime \prime }\left(\omega \right)$ obtained in slip-spring model simulations of star polymers with arm length N=36 without constraint release (blue filled circles) and experimental measurements of polyisoprene 6-arm star probes with arm molecular weight $M_{a}=24k$ in long linear PI chain matrix (red open circles) [47]. The slip-spring data have been shifted by multiplying a factor of $6\times 10^{5}$ in frequency ω and 0.075 in $\epsilon^{\prime \prime }$, respectively.

The coincidence of the frequency dependence of the dielectric (end-to-end relaxation) and viscoelastic losses in systems with quenching CR effects has been shown experimentally by Matsumiya et al. using polyisoprene star probes in long linear chain matrices [47]. In Figure 11, we compare our slip-spring model simulation results on the dielectric loss of star polymers with arm length N=36 with the experimental data ($\epsilon_{1,b}^{\prime \prime }$) in Figure 5d of Reference [47], where the 6-arm PI star probes with arm molecular weight $M_{a}=24k$ are dispersed in a matrix of PI chains with molecular weight 1.1M. The reasonably good agreement between the two sets of data indicates that the slip-spring model without CR can essentially capture the CR quenching behavior observed in experiments. It can thus be used together with slip-spring/slip-link [31,48,49] and bead-spring molecular dynamics [31,50,51,52] methods for bulk systems to provide microscopic understanding of constraint release mechanisms, in a similar manner to that done in experiments [47]. For the comparison in Figure 11, we noticed that there is a small difference in the numbers of entanglements per arm as used in experiments ($Z=M_{a}/M_{e}\approx 4.8$ calculated using $M_{e}=5k$ for PI) and slip-spring simulations ($Z=N/N_{e}\approx 6.3$ estimated using ${\tilde{N}}_{e}\approx 5.7$). This again can be attributed to the variation in the $N_{e}$ values determined from mapping simulation results to different experimental observables.

In a star polymer system without CR, the survival probability of a slip-link or correspondingly tube segment at a fractional distance *s* from the mean equilibrium position of the arm free end at time *t* can be calculated using the FP time distribution f(s,τ) [2,31],

(12) $$\begin{array}{c} \psi \left(s,t\right)=\int_{t}^{+\infty }f\left(s,\tau \right)d\tau . \end{array}$$

If the primitive path is considered to be continuous in space, the normalized tube survival function μ(t) can be obtained by integrating over the tube segment survival probability ψ(s,t) along the PP

(13) $$\begin{array}{c} \mu \left(t\right)=\int_{0}^{1}\psi \left(s,t\right)ds. \end{array}$$

Based on the analysis in Section 5, the FP time distributions generally follow the exponential form for tube segments or slip-links close to the branch points so that ψ(s,t) can be approximated by $\exp(-t/\tau_{ncr}\left(s\right))$ where the mean FP time $\tau_{ncr}\left(s\right)$ is calculated using Equation (9) with the simulation results on f(s,τ). It follows that

(14) $$\begin{array}{c} \mu \left(t\right)\approx \int_{0}^{1}\exp\left(-\frac{t}{\tau_{ncr}\left(s\right)}\right)ds. \end{array}$$

**Figure 12:**
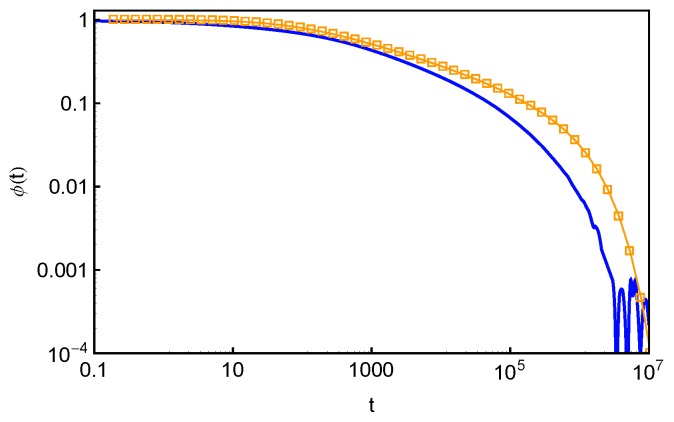
Normalized tube survival function μ(t)/μ(0) (symbols) calculated using Equation (14) with simulation results on $\tau_{ncr}\left(s\right)$ and arm end-to-end vector correlation function ϕ(t) (solid line) obtained from the same set of simulations for the system with star arm length N=30.

The normalized μ(t)/μ(0) results calculated using Equation (14) and f(s,τ) obtained in slip-spring model simulations are presented in Figure 12 for the star arms with length N=30. The arm end-to-end vector correlation function ϕ(t) obtained in the same set of simulations is also included for comparison.

**Figure 13:**
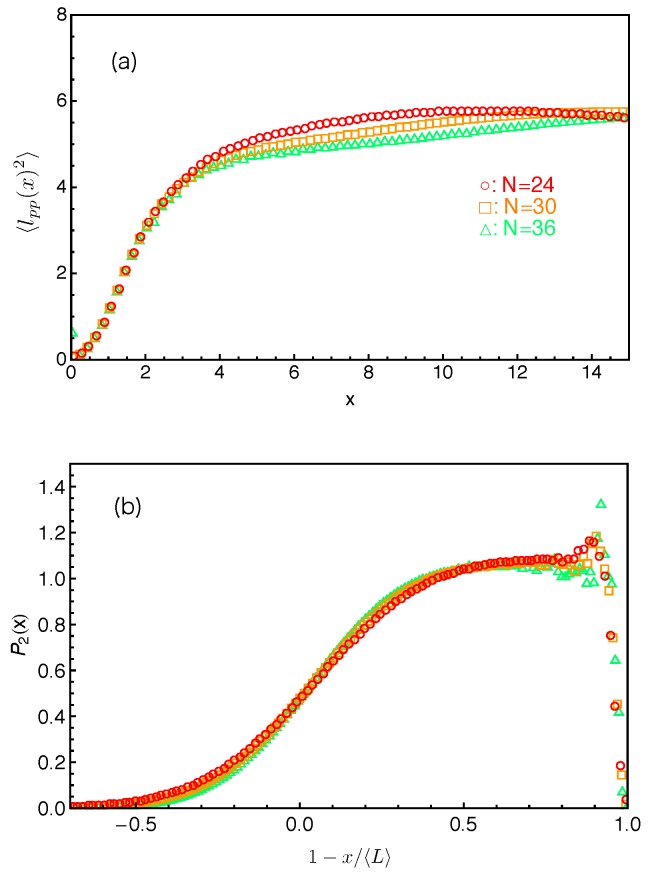
(**a**) Mean squared primitive path segment lengths, $l_{PP}^{2}\left(x\right)$, released by the destruction of slip-slinks at a primitive path distance *x* away from the branch point; (**b**) probability $P_{2}\left(s\right)$ of finding a slip-link at a fractional PP distance s=1−x/〈L〉 away from the mean equilibrium position of the arm free end.

In the Doi–Edwards theory, the end-to-end vector correlation function, $\phi \left(t\right)\equiv \frac{\langle {\vec{R}}_{ee}\left(t\right){\vec{R}}_{ee}\left(0\right)\rangle }{\langle R_{ee}^{2}\left(0\right)\rangle }$, of a polymer chain in a fixed network is equal to the normalized survival function μ(t)/μ(0) of its original confining tube [2]. Here, ${\vec{R}}_{ee}$ is the chain end-to-end vector. The ϕ(t) and normalized μ(t) results in Figure 12, however, do not show the expected agreement. Such discrepancy originates from the fact that Equation (14) is valid only if the FP time distribution f(s,τ) is exponential at all *s* values and the tube is continuous such that the deletion of each entanglement releases an original tube segment of the same length. Both conditions are not fully satisfied in the discrete slip-spring model for star polymers. The assumed exponential form of f(s,τ) does not apply for the slip-links at relatively small *s* values or farther away from the branch points. On the other hand, the tube or primitive path segment lengths released by destructing slip-links next to the branch points do not follow the assumed uniform distribution. As sketched in Figure 1, the destruction of a slip-link located at a primitive path distance *x* from the branch point by the arm free end at time *t* will release the PP segment of length $l_{PP}\left(x\right)$ in between itself and the nearest inner neighboring slip-link (or branch point) along the arm. By ensemble average, the mean squared end-to-end distance of the surviving part of the original primitive path at time *t* is reduced by an amount of $\langle l_{PP}^{2}\left(x\right)\rangle$ [2]. The simulation results on $\langle l_{PP}^{2}\left(x\right)\rangle$ are shown in Figure 13a for the systems with different arm lengths. It can be seen that $\langle l_{PP}^{2}\left(x\right)\rangle$ first increases quadratically with *x* and then approaches a plateau at x>a where the tube step length $a\approx {\tilde{N_{e}}}^{1/2}b_{1D}$. The deviation from the theoretically assumed uniform profile occurs for the innermost slip-links, which are at distances x≤a from the branch points. The primitive path lengths held in between them and the branch points are simply proportional to *x*, giving $\langle l_{PP}^{2}\left(x\right)\rangle \approx x^{2}$. The uncertainty in determining the terminal arm retraction time is also associated with the distribution of the innermost PP segment lengths to be released by the arm free ends.

**Figure 14:**
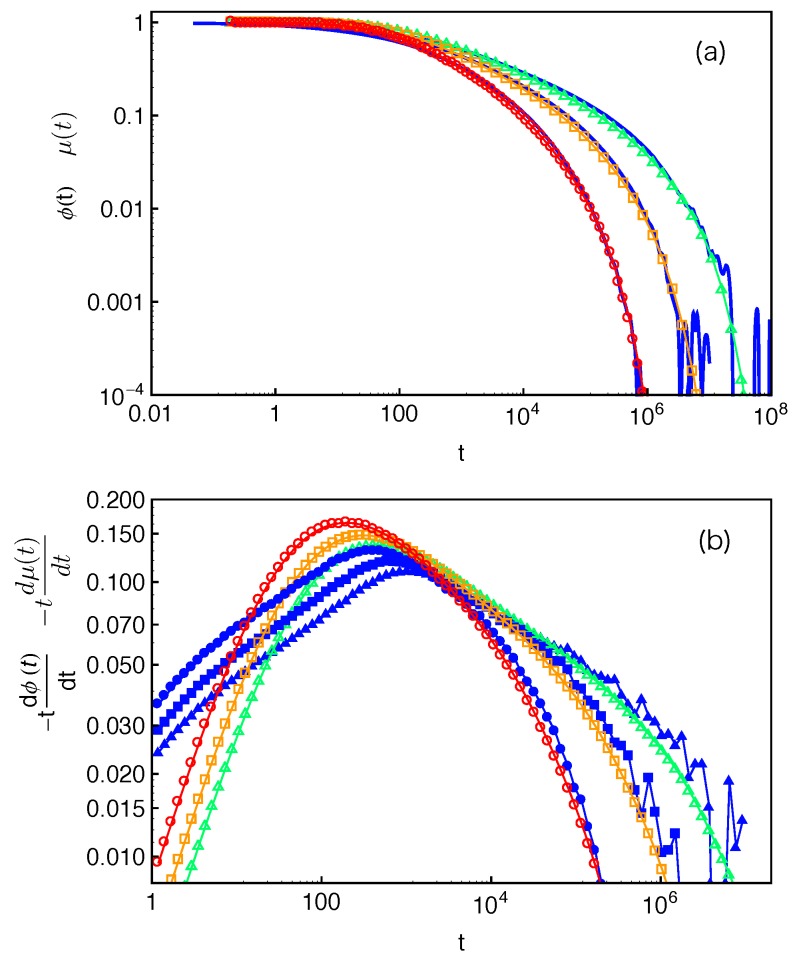
(**a**) Comparison of the tube survival functions μ(t) (symbols) calculated using Equation (15) and the arm end-to-end vector correlation functions ϕ(t) (lines) directly measured in the slip-spring simulations for the systems with arm lengths N=24, 30 and 36 from left to right; (**b**) derivatives $-t\frac{d\mu (t)}{dt}$ (open symbols) and $-t\frac{d\phi (t)}{dt}$ (solid symbols) of the functions given in (**a**).

From a numerical point of view, the tube survival function μ(t) can be calculated more accurately by using the simulation results on both the PP segment lengths $l_{PP}\left(x\right)$ held by the slip-links located at the position *x* from the branch points along the primitive path and the probability $P_{2}\left(x\right)$ of finding an original slip-link at *x*. The simulation results on $P_{2}\left(x\right)$, or more precisely $P_{2}\left(s\right)$, are plotted in Figure 13b with respect to the fractional PP distance s(=1−x/〈L〉) of the slip-links from the mean equilibrium positions of the arm free ends for the systems studied in Figure 13a. All sets of $P_{2}\left(s\right)$ data drop on the same universal curve, which shows a broad distribution around s=0 (x=〈L〉) due to the large fluctuations of the arm PP lengths around their mean value 〈L〉 and becomes constant at intermediate and small *s* values except for being very close to the branch points. The tube survival function μ(t) can then be calculated as

(15) $$\begin{array}{c} \mu \left(t\right)\approx \frac{\int l_{PP}^{2}\left(s\right)P_{2}\left(s\right)\exp\left(-\frac{t}{\langle \tau (s)\rangle }\right)ds}{\int l_{PP}^{2}\left(s\right)P_{2}\left(s\right)ds}, \end{array}$$

which is normalized such that μ(t=0)=1. We can see that the denominator equals to a〈L〉. The obtained μ(t) results are presented in Figure 14a and show very good agreement with the directly measured arm end-to-end vector correlation functions ϕ(t). To reveal more subtle differences and regimes, it is particularly useful to compare the dimensionless derivatives of the two functions, $-t\frac{d\phi (t)}{dt}$ and $-t\frac{d\mu (t)}{dt}$. As shown in Figure 14b, for each given arm length, the two derivatives have reasonably good agreement at large time scales. The discrepancy at early times is again related to the use of the exponential approximation instead of the numerical results on the FP time distribution f(s,τ) at small *s* values.

The results in Figure 14 demonstrate the applicability of Equation (15) for estimating the tube survival function μ(t). However, the requirement of inputting simulation data on $l_{PP}^{2}\left(s\right)$ and $P_{2}\left(s\right)$ strongly limits its prediction power. Based on the universal behavior of $l_{PP}^{2}\left(x\right)$ and $P_{2}\left(x\right)$ observed in Figure 13, we propose the following empirical expressions to approximate these quantities. First, we assume that the slip-links are uniformly distributed along the primitive path from the branch point to the mean equilibrium position of the arm free end, which gives a step or indicator function for $P_{2}\left(x\right)$,

(16) $$\begin{array}{c} P_{2}\left(x\right)=\left\{\begin{array}{cc} 1/\langle L\rangle , & \;0\leq x\leq \langle L\rangle ,\\ 0, & \;otherwise. \end{array}\right. \end{array}$$

**Figure 15:**
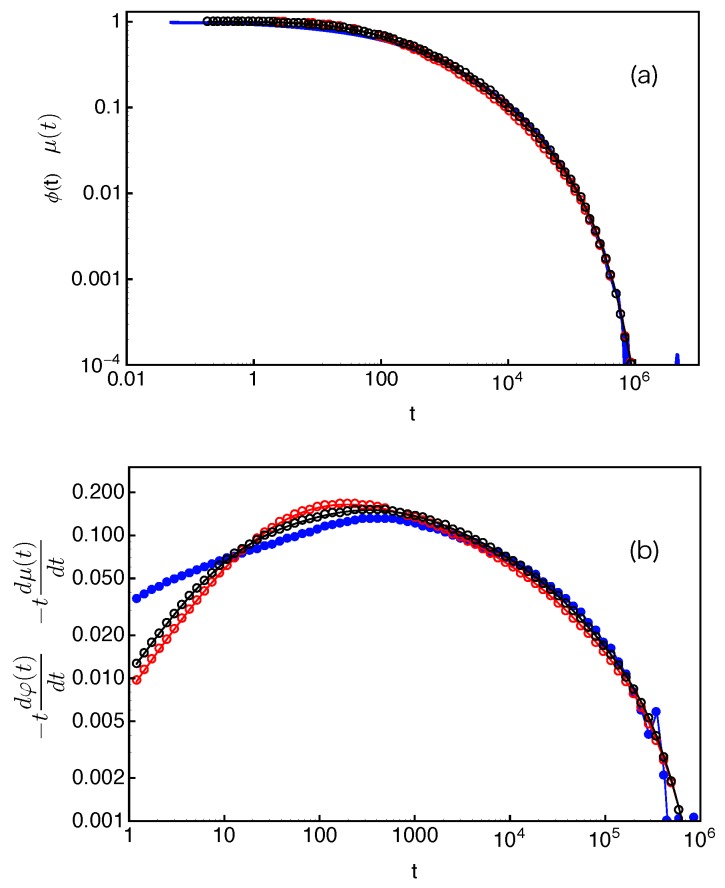
(**a**) Comparison of the tube survival functions μ(t) (black symbols) calculated by substituting Equations (16) and (17) into Equation (15) and the arm end-to-end vector correlation functions ϕ(t) (blue lines, same as the one in Figure 14a) directly measured in the slip-spring simulations for the system with arm length N=24; (**b**) derivatives $-t\frac{d\mu (t)}{dt}$ (open black symbols) and $-t\frac{d\phi (t)}{dt}$ (solid blue symbols) of the functions given in (**a**). Red symbols are the same plots as those in Figure 14.

Secondly, we describe the simulation data on $l_{pp}^{2}\left(x\right)$ with an empirical function

(17) $$\begin{array}{c} l_{PP}^{2}\left(x\right)={\left(\frac{1}{x^{-2\beta }+C^{-\beta }}\right)}^{1/\beta }, \end{array}$$

where the two fitting parameters are found to be C=4.87 and β=1.69. The value of *C* corresponds to the plateau magnitude of $l_{pp}^{2}\left(x\right)$. By substituting Equations (16) and (17) into Equation (15), we calculate μ(t) and its derivative $-t\frac{d\mu (t)}{dt}$ for the systems studied in Figure 14. As shown in Figure 15, the results agree with ϕ(t) and $-t\frac{d\phi (t)}{dt}$ very well. Equations (15)–(17) or similar formulae may also be used in other discrete simulation models for describing the relaxation correlation functions.

The tube survival probability can also be calculated from the probability density that a randomly selected entanglement has a lifetime *t*, as has been used in the single-chain slip-spring and molecular dynamics simulations of monodisperse symmetric star polymer melts [31], where this probability is termed as $P_{ent}\left(t\right)$. In the absence of constraint release, all entanglements or slip-links are destroyed by the retracting arm ends, the $P_{ent}\left(t\right)$ results will be consistent with μ(t) calculated with Equation (15) and so with the end-to-end vector correlation function ϕ(t). Since recent theoretical models on branch polymer dynamics, such as the Milner–McLeish and large deviation theories, are mainly based on the calculation of first-passage times of entanglements, we will limit our discussions on μ(t) in the current work.

## 7. Conclusions

In this work, we perform single-chain slip-spring model simulations to investigate arm retraction dynamics of entangled star polymers in the absence of constraint release. In the mesoscopic slip-spring model, entanglements are represented by slip-links that can only be created and destroyed from the arm free ends. The primitive path of a confined star arm is constructed by connecting either the successive anchor points or the mean positions of the slip-links with straight lines. The distributions of the primitive path lengths *L* are calculated for the systems with different arm lengths and analyzed to extract tube theory parameters, including the key parameter of entanglement molecular weight $N_{e}$. It is found that the PP lengths in between the innermost slip-links and the arm free ends closely follow the Gaussian distribution as expected from the standard tube theory, which also supports the quadratic form of the entropic energy barrier, also called arm retraction potential, used in various tube-based theories. The asymptotic $N_{e}$ value (5.7) obtained from the static PP analysis are consistent with that found in a previous work obtained from mapping slip-spring simulation results on linear viscoelastic properties to tube model predictions [55]. Due to the discrete feature of the slip-spring model, the tube or PP segments in between the innermost slip-links and the branch points are released entirely when the innermost slip-links are destructed by the retracting arm free ends. As a consequence, the entropic energy barrier close to the branch points and accordingly the terminal arm relaxation times determined from simulations based on the slip-spring model and probably also other discrete models are different from those predicted by the tube theories where the tubes are assumed to be continuous such that the arm free ends can retract all the way to the branch points.

We then calculate the first-passage times for destructing the slip-links or entanglements on the fly during the simulation runs. The FP time distributions f(s,τ) follow the exponential form if the slip-links or entanglements are close to the branch points, but become non-exponential for the entanglements at small fractional distances *s* from the mean equilibrium positions of the arm free ends. Simulation results on the mean FP times $\tau_{ncr}\left(s\right)$ are fitted to the prediction of a large deviation theory of one-dimensional Rouse chain. The extracted $N_{e}$ values show a nearly linearly dependence on the inverse arm length. The asymptotic $N_{e}$ value obtained from this dynamic analysis is close to but slightly lower than that given by the primitive path analysis, which is presumably related to the different primitive path portions used for the static and dynamic analyses, namely excluding or including the PP segments in between the innermost slip-links and the branch points.

Following the suggestions of tube theories, the FP time distributions f(s,τ) are used to determine the tube survival functions μ(t) that are subsequently compared with the arm end-to-end vector correlation functions ϕ(t) directly measured in the simulations. The μ(t) results calculated based on the tube theory assumptions of continuous tube and exponential FP time distributions at all *s* values do not show the agreement with ϕ(t) as would have been expected from the Doi–Edwards theory. The reason lies in the fact that both assumptions are not fully satisfied in the discrete slip-spring model. By taking into account the nonuniform distribution of the PP segments released by the destruction of slip-links at different locations along the primitive path, especially those next to the branch points, we are able to predict μ(t) using only the mean FP times $\tau_{ncr}\left(s\right)$ that are in reasonably good agreement with ϕ(t) for the arm lengths we studied as well as experimental results on dielectric relaxation obtained from star probes blended in long chain matrix. As a final remark, we note that the dynamics of entangled star polymer melts are determined by both the arm retraction and constraint release mechanisms. A detailed study of CR effects in star polymer systems through multiscale simulation approaches can be found in our recent publication [31].

## Figures and Tables

**Table 1:** Tube theory parameters estimated from slip-spring simulations by using two different constructions of the primitive path, i.e., positions of anchor points and average slip-link (SL) positions.

PP Construction	$\langle {\vec{v}}_{1}^{2}\rangle$	$\langle |{\vec{v}}_{1}|\rangle$	a(N→+∞)	$N_{e}\;\left(a^{2}/{b_{3D}}^{2}\right)$	$b_{1D}\;\left(N\;\to \;+\infty \right)$	${\mathrm{ab}}_{1D}$	$\tilde{N_{e}}\;\left(a^{2}{b_{1D}}^{2}/{b_{3D}}^{4}\right)$
Anchor points	4.19	1.77	2.01	4.02	1.17	2.35	5.52
Average SL positions	3.37	1.53	2.59	6.71	0.94	2.43	5.90

## Data Availability

The simulation data reported in this work are available upon request from the corresponding authors.
